# Detection of a streptogramin A O-acetyltransferase gene (*vatD*) in the chromosome of *Clostridium botulinum* isolated from infants in the United States

**DOI:** 10.1128/aem.00090-25

**Published:** 2025-07-22

**Authors:** Ana Rafaela Kruemmel, Jessica L. Halpin, Victoria M. Foltz, Janet K. Dykes, Carolina Lúquez

**Affiliations:** 1 National Botulism Laboratory, Enteric Diseases Laboratory Branch, Centers for Disease Control and Prevention 1242, Atlanta, Georgia, USA; Norwegian University of Life Sciences, Ås, Norway

**Keywords:** infant botulism, antimicrobial resistance genes, *Clostridium botulinum*, streptogramin A, *bont* gene

## Abstract

Infant botulism, the most common form of botulism in the United States, occurs when botulinum toxin-producing clostridia colonize the infant’s gut and produce botulinum neurotoxin (BoNT). This condition is treated with botulinum antitoxin and supportive care; antibiotics are avoided due to the risk of worsening symptoms by promoting toxin release. Consequently, antimicrobial resistance genes (ARGs) are rarely investigated in *Clostridium botulinum* strains. During 2020 through 2023, 226 *C*. *botulinum* isolates associated with infant botulism were sequenced in our laboratory using whole-genome sequencing (WGS) technologies. *In silico* analysis of the WGS data revealed that 15% of these isolates (*n* = 36) carried a *vatD* gene variant, which is associated with resistance to streptogramin A antibiotics. In this study, we describe the molecular characterization of 36 isolates that harbor the *vatD* gene. To the best of our knowledge, this is the first report of the detection of the *vatD* gene in the chromosome of *C. botulinum* recovered from infants in the United States.

**IMPORTANCE:**

The continuous expansion of whole-genome sequencing (WGS) technologies, combined with refined data analysis tools, has enabled an in-depth analysis of pathogens, allowing a thorough characterization and comprehension of the genomic diversity of *C. botulinum*. In the present study, we demonstrate how the implementation of WGS into laboratory surveillance workflow allowed the detection of an antimicrobial resistance gene, the *vatD* gene, in the chromosome of *C. botulinum* strains recovered from infants. To the best of our knowledge, this is the first study to report the detection of the *vatD* gene in non-enterococci isolates recovered from clinical samples in the United States.

## INTRODUCTION

*Clostridium botulinum* is a Gram-positive, spore-forming, anaerobic bacterium that is capable of producing one of the most dangerous neurotoxins, botulinum neurotoxin (BoNT). BoNTs are classified into seven immunologically distinct serotypes (BoNT/A to /G), by their ability to neutralize specific polyclonal antisera (1) and further characterized into more than 40 subtypes, based on the amino acid sequence variation (>2.6%) identified within the botulinum neurotoxin (bont) gene (2). Toxicity levels can vary between the different serotypes. BoNT/A, B, E, and F are the main causative agents of human botulism; serotypes A and B are the most prevalent in the United States (US) (3).

The clinical manifestation of botulism occurs when BoNTs irreversibly bind to presynaptic cholinergic receptors, blocking the release of acetylcholine. This results in the failure of neuromuscular transmission, flaccid paralysis, and in rare cases, death. The disease has five well-defined types of manifestation: infant botulism, foodborne botulism, wound colonization botulism, iatrogenic botulism, and adult intestinal colonization (3). Infant botulism (IB), the most common form of botulism in the US (4), occurs when spores of *C. botulinum* are ingested by infants (aged <1 year), resulting in germination and multiplication of the bacterium within the intestinal tract and the production and release of BoNTs *in situ* (3). In the US, IB treatment consists of the administration of BabyBIG botulinum antitoxin (5) and supportive care, which may include mechanical ventilation and enteral feeding. Antimicrobial therapy is not recommended due to the increased risk of lysis of *C. botulinum* vegetative cells, which might exacerbate the release of BoNT, worsening the botulism symptoms (6–8).

Infant botulism is confirmed by the detection of BoNT in the stool or by the detection of a viable toxin-producing *C. botulinum* isolate in the stool. The Centers for Disease Control and Prevention (CDC) conducts laboratory confirmation and surveillance of botulism cases in the US. Annually, the CDC National Botulism Laboratory (NBL) investigates an average of 50 suspected cases of IB and performs laboratory testing on clinical samples; this includes direct detection of BoNT and isolation of *C. botulinum* and other BoNT-producing clostridia from clinical samples (9). In addition, NBL conducts molecular surveillance using whole-genome sequencing (WGS) technologies to characterize isolates from confirmed cases, including isolates submitted by national and international partners.

Because antibiotics may worsen IB symptoms by either increasing the availability of BoNT in the intestine due to cell death or by altering the intestinal microbiota and favoring *C. botulinum* overgrowth (8), detection of antimicrobial susceptibility has limited clinical relevance in IB cases, and antimicrobial susceptibility tests (AST) are not performed during routine botulism laboratory confirmation. Hence, there is limited data about antibiotic resistance in *C. botulinum* strains. The implementation of WGS technologies in the laboratory workflow, however, has expanded detection capabilities to include additional analyses that are not part of the routine, including the detection of antimicrobial resistance genes (ARG). For instance, Mazuet et al. (10) described the detection of a β-lactamase gene cluster in several *C. botulinum* strains using WGS analysis, and in 2022, Ma et al. (11) reported that 74% of the isolates sequenced in their study harbored the multidrug resistance genes CMY-15 and lmrB.

From 2020 through 2023, a total of 226 isolates associated with IB cases in the US, recovered either from current laboratory investigations or from NBL’s historical inventory, were sequenced and analyzed in our laboratory. *In silico* screening of ARGs revealed that 15% of the isolates (*n* = 36) harbored a *vatD* gene*,* which encodes the acetylation of streptogramin A antibiotics (12) as a mechanism of resistance.

Streptogramin antibiotics are composed of two chemically distinct depsipeptides, types A and B, and include compounds such as virginiamycin and quinupristin‐dalfopristin (Q/D; Synercid) that can act synergistically by inhibiting protein synthesis in aerobic and anaerobic Gram-positive bacteria (13). Several ARGs have been associated with mechanisms of resistance to streptogramin antibiotics, including the *vatD* gene (14) and, at a lower prevalence, the presence of the *vatD* gene has been detected exclusively in the plasmid of streptogramin-resistant enterococci isolates recovered from both human and non-human sources in Europe (15–17) and in animals and environmental sources in the US (18).

Enterococci are ubiquitous pathogens, frequently found in the same reservoirs as clostridial species, with remarkable capabilities to acquire and transfer virulence factors mainly through horizontal gene transfer (19). Likewise, in recent years, a growing number of studies have shown *C. botulinum* genomic plasticity, particularly by the detection of *bont* genes homologs in strains within (20) and outside of the Clostridium genus (21, 22), including the detection of a *bont-*like gene in an *E. faecium* strain (23). In the present study, we used WGS data to conduct a comparative genomic and phylogenetic analysis of the Clostridium isolates harboring a *vatD* gene homologous to that found in *E. faecium*. The findings presented in this descriptive study might provide insights into a possible bidirectional relationship among those species.

## MATERIALS AND METHODS

### Bacterial strains

The 36 isolates analyzed in this study were either recovered from fecal specimens sent to the CDC for confirmation of US infant botulism cases or as part of a bulk shipment of historical strains from State Public Health Laboratories (SPHL) (Table 1).

**TABLE 1:** Summary of the 36 strains harboring the *vatD* gene variant characterized in this study

Strain	State	Origin	Source type	Toxin subtype	Sequence type (ST-)	Accession
CDC64016B1	AR	Case Investigation	Stool	B1	100	SRR30606176
CDC64159	TX	SPHL^*a*^	Isolate	B1	100	SRR30606175
CDC64160	TX	SPHL^*a*^	Isolate	B1	119	SRR30606164
CDC64161	TX	SPHL^*a*^	Isolate	B1	100	SRR30606153
CDC64163	TX	SPHL^*a*^	Isolate	B1	100	SRR30606146
CDC64165	TX	SPHL^*a*^	Isolate	B1	100	SRR30606145
CDC64167	TX	SPHL^*a*^	Isolate	B1	100	SRR30606144
CDC64176	TX	SPHL^*a*^	Isolate	B1	100	SRR30606143
CDC64181	TX	SPHL^*a*^	Isolate	B1	100	SRR30606142
CDC64182	TX	SPHL^*a*^	Isolate	B1	100	SRR30606141
CDC64183	TX	SPHL^*a*^	Isolate	B1	100	SRR30606174
CDC64185	TX	SPHL^*a*^	Isolate	B1	100	SRR30606173
CDC64187	TX	SPHL^*a*^	Isolate	B1	100	SRR30606172
CDC64190	TX	SPHL^*a*^	Isolate	B1	118	SRR30606171
CDC64192	TX	SPHL^*a*^	Isolate	B1	100	SRR30606170
CDC64193	TX	SPHL^*a*^	Isolate	B1	115	SRR30606169
CDC64195	TX	SPHL^*a*^	Isolate	B1	100	SRR30606168
CDC64197	TX	SPHL^*a*^	Isolate	B1	100	SRR30606167
CDC64295E1	AR	Case investigation	Stool	B1	128	SRR30606166
CDC67234	TX	SPHL^*a*^	Isolate	B1	119	SRR30606165
CDC75010PL1	NC	Case investigation	Stool	B1	123	SRR30606163
CDC75088T1	AZ	Case investigation	Stool	B1	134	SRR30606162
CDC75119PL2	NC	Case investigation	Stool	B1	176	SRR30606161
CDC76028H1	LA	Case investigation	Stool	B1	100	SRR30606160
CDC76029B1	AR	Case investigation	Stool	B1	115	SRR30606159
CDC76090	MN	SPHL^*a*^	Stool	B1	100	SRR30606158
CDC76110	MN	SPHL^*a*^	Stool	B1	146	SRR30606157
CDC76122B1	MO	Case investigation	Stool	B1	100	SRR30606155
CDC76190T1	LA	Case investigation	Stool	B1	100	SRR30606154
CDC76250PL1	AR	Case investigation	Stool	B1	100	SRR30606152
CDC77012B1	LA	Case investigation	Stool	B1	159	SRR30606151
CDC77067H1	AR	Case investigation	Stool	B1	100	SRR30606150
CDC77171B1	TX	Case investigation	Stool	B1	100	SRR30606149
NT76117PL3	AR	Case investigation	Stool	NT	N/A	SRR30606156
NT76259H2	MO	Case investigation	Stool	NT	100	SRR30606148
NT77026E1	TX	Case investigation	Isolate	NT	N/A	SRR30606147

^*a*^ State Public Health Laboratory Bulk shipment; NT—non-toxigenic; N/A— not applicable.

Fecal samples from botulism case laboratory investigations were examined for the presence of BoNT-producing clostridia by standard methods (9). Briefly, an aliquot of either direct fecal sample or previous enriched cultures was streaked by quadrant isolation into McClung-Toabe egg yolk agar (EYA) or botulism selective medium (BSM) plates and incubated under anaerobic conditions at 35°C ± 2°C for 2–4 days. Single lipase-positive colonies were picked and inoculated into chopped meat glucose starch broth (CMGS) (Remel; Lenexa, KS, USA). These were incubated anaerobically for 3–7 days at 35°C ± 2°C.

Transferred strains from SPHL were shipped frozen (−20°C ± 2°C) in brain heart infusion (BHI) medium with 20% glycerol and immediately inoculated into CMGS broths. Broth cultures were incubated anaerobically at 35°C ± 2°C for 3–7 days, and an inoculum of ~0.5 mL was streaked for isolation onto EYA plates. Plates were incubated anaerobically at 35°C ± 2°C for 48 h and inspected for purity.

All isolated single colonies were inoculated into trypticase peptone glucose yeast extract broth (TPGY) (Remel, Lenexa, KS, USA) and incubated anaerobically at 35°C ± 2°C for 16–18 h prior to genomic DNA extraction.

### Genomic DNA extraction and whole-genome sequencing

In compliance with human subjects research protocol (CDC Institutional Review Board, protocol number 6911), all strains used in this study were de-identified prior to DNA extraction. Genomic DNA was extracted using a modified MasterPure complete DNA and RNA purification kit (Lucigen, Middleton, WI, USA) (24). DNA quality and quantification were assessed using the NanoDrop 2000 (NanoDrop Technologies, Wilmington, DE, USA) and the Qubit 4 (Invitrogen, Waltham, MA, USA) fluorometer high-sensitivity assay following the manufacturer’s instructions.

DNA libraries were generated using the Illumina DNA Prep kit and sequenced using either a MiniSeq System (2 × 150 bp chemistry) or a MiSeq System (2 × 250 bp chemistry) (Illumina, San Diego, CA, USA), according to the manufacturer’s instructions.

### Genome assembly, quality control, and annotation

Paired-end raw reads were processed using the Bactopia pipeline version 3.0.0 (25) for data quality assessment, assembly, annotation, call of 7-gene multilocus sequence type (MLST) (see Table S1 for quality metrics raw data), and identification of antimicrobial resistance genes. All reads were processed using a modified parameter (bactopia --samples vatD2023.txt --run_name vatD2023 -profile singularity --shovill_assembler spades --min_contig_len 500 --species “Clostridium botulinum” --genome_size 4000000 --cleanup_workdir). Potential new MLST profiles were verified by querying assemblies against the *C. botulinum* PubMLST database (26, 27), and new alleles and sequence types (STs) were submitted to the publicly available MLST database (http://pubmlst.org/cbotulinum/). Completeness and the presence of contaminants in the assemblies were verified using the module checkM (28).

### Toxin subtype identification and plasmid prediction

*bont* gene subtype was determined using a combination of the GAMMA v.2.1 (29) module within the Bactopia toolkit and an in-house-built database of *bont* gene subtypes and protein accessory genes using nucleotide sequences retrieved from the National Center for Biotechnology Information (NCBI) GenBank database (30). The putative location of the *bont* gene on either the chromosome or within a plasmid was predicted using the module MOB-suite v.3.1.4 (31). The extracted putative plasmid sequences were further analyzed, confirmed with blastn v.2.14.1, last accessed April 2024 (32), and visualized using BRIG v.0.95-dist (33).

### Antimicrobial resistance gene and mobile elements analysis

By default, Bactopia screens all the draft genome assemblies for the presence of known antimicrobial resistance genes (ARG) using the NCBI Antimicrobial Resistance Gene Finder (AMRFinderPlus) tool v.3.11.11 (34). To confirm the presence of the ARGs, assemblies were inspected using ABRicate v.1.0.1 (https://github.com/tseemann/abricate) with multiple antimicrobial resistance gene databases, such as ResFinder (35), CARD (36), and MEGARES (37). In addition, assemblies were scanned for plasmids and mobile genetic elements (MGE) using PlasmidFinder v.2.1 (38) and MobileElementsFinder v.1.0.3 (39), respectively.

### Analysis of the *vatD* gene

Annotated sequences of the draft genomes were screened using Geneious Prime v.2020.0.5 for visualization of the predicted *vatD* gene within the strains. Annotated *vatD* nucleotide sequences were extracted using the function “Extract Regions” and subsequently translated to protein sequences, using the default settings. Multiple sequence alignment from nucleotide and amino acid sequences was performed using the Clustal Omega plugin within Geneious Prime. Furthermore, the annotated genomes were inspected for the presence of a genomic island (GI) using IslandViewer4 (40).

Predicted protein sequences were screened against the UniProt database using BLAST (https://www.uniprot.org/blast), and an alignment and comparison of enzymatic active site presence was performed against the protein sequence P50870 (EC:2.3.1:Streptogramin A acetyltransferase, https://www.uniprot.org/uniprotkb/P50870/entry), the sequence with the highest percentage identity, lowest e-value, and with manual assertion inferred by UniProtKB curator.

Pathogen Detection web browser at NCBI (https://www.ncbi.nlm.nih.gov/pathogens/) was screened for genome sequences of *Clostridium botulinum* isolates encoding the *vatD* gene (search terms: AMR_genotype: vatD and taxgroup_name: “Clostridium botulinum,” last accessed November 8, 2023). In all, 19 genomes were downloaded, 18 strains from China from both clinical and environmental sources, and 1 strain from Argentina from a foodborne case (Table S2). The *vatD* sequences from the genome of those strains were extracted using an in-house script blast-and-extract_v3.pl and included in a multiple sequence comparison and construction of a maximum likelihood tree using MEGA X (41). Tree annotation and visualization were done using iTOL v.6.9 (42).

### Gene cluster alignment, schematic gene map representation, and visualization

The *vatD* gene sequences and flanking regions were extracted from the assembled files using an in-house script blast-and-extract_v3.pl. A global alignment and comparison of the *vatD* gene cluster (~4 Kb nucleotides) was performed using clinker, and a gene map visualization was completed with clustermapj (43).

### Comparative genome analysis

The 36 *vatD*+ *Clostridium* isolates genomes were mapped to the Okra B1 reference genome (accession: NC_010516.1) using Snippy v.4.6.0 (bactopia --wf snippy --bactopia bactopia --reference okraB1.gb -profile singularity). The strain Okra B1 was selected as a reference based on a preliminary analysis using a combination of Bactopia submodules Mashtree v.1.2.0, fastANI v.1.33, and ncbi-genome-download v.0.3.3. The resulting core genome alignment was employed to build a maximum likelihood phylogenetic tree with IQ-Tree v.2.2.0.3 and visualized using iTOLv.6.9 (42). A resulting pairwise distance matrix between the isolates was generated as part of the workflow, using snp-dists v.0.8.2.

A comparative pangenome analysis between the strains from this study and the *vatD*+ strains from China (*n* = 18) and Argentina (*n* = 1) was performed using Panaroo v.1.5.0 (44). The output files were post-processed to estimate the openness of the pangenome using the script pan_genome_analysis.py (https://github.com/vsmicrogenomics/PanGenomeAnalysisTool) under the fundamentals of Heaps’ Law (45). In addition, the script roary_ploy.py (https://github.com/sanger-pathogens/Roary/tree/master/contrib/roary_plots) was used to plot a pie chart with distribution between the core and accessory genes and a phylogenetic tree with a presence/absence matrix.

## RESULTS

### Infant botulism strains

Of the 36 strains further characterized in this study, 33 harbored the *bont*/B1 gene. Three isolates were identified as non-toxigenic (NT) by routine laboratory toxin detection testing, and the absence of the *bont* gene cluster was confirmed by WGS analysis of the draft genome assemblies. Isolates and stool samples were originally submitted from seven different US states, with most of the isolates being originally from Texas (TX, *n* = 20), followed by Arkansas (AR, *n* = 6), and with states of Louisiana (LA), Missouri (MO), Minnesota (MN), and North Carolina (NC) being associated with three or less isolates each. Eight different MLST sequence types (ST) were identified, and four of these were new (ST-134, ST-146, ST-159, and ST-176). The most prevalent was ST-100 (*n* = 23), followed by ST-115 and ST-119 (*n* = 2, each), with the remaining STs being represented by a single isolate. In addition, two NT isolates were non-typable by the 7-gene MLST (Table 1).

**Fig 1:**
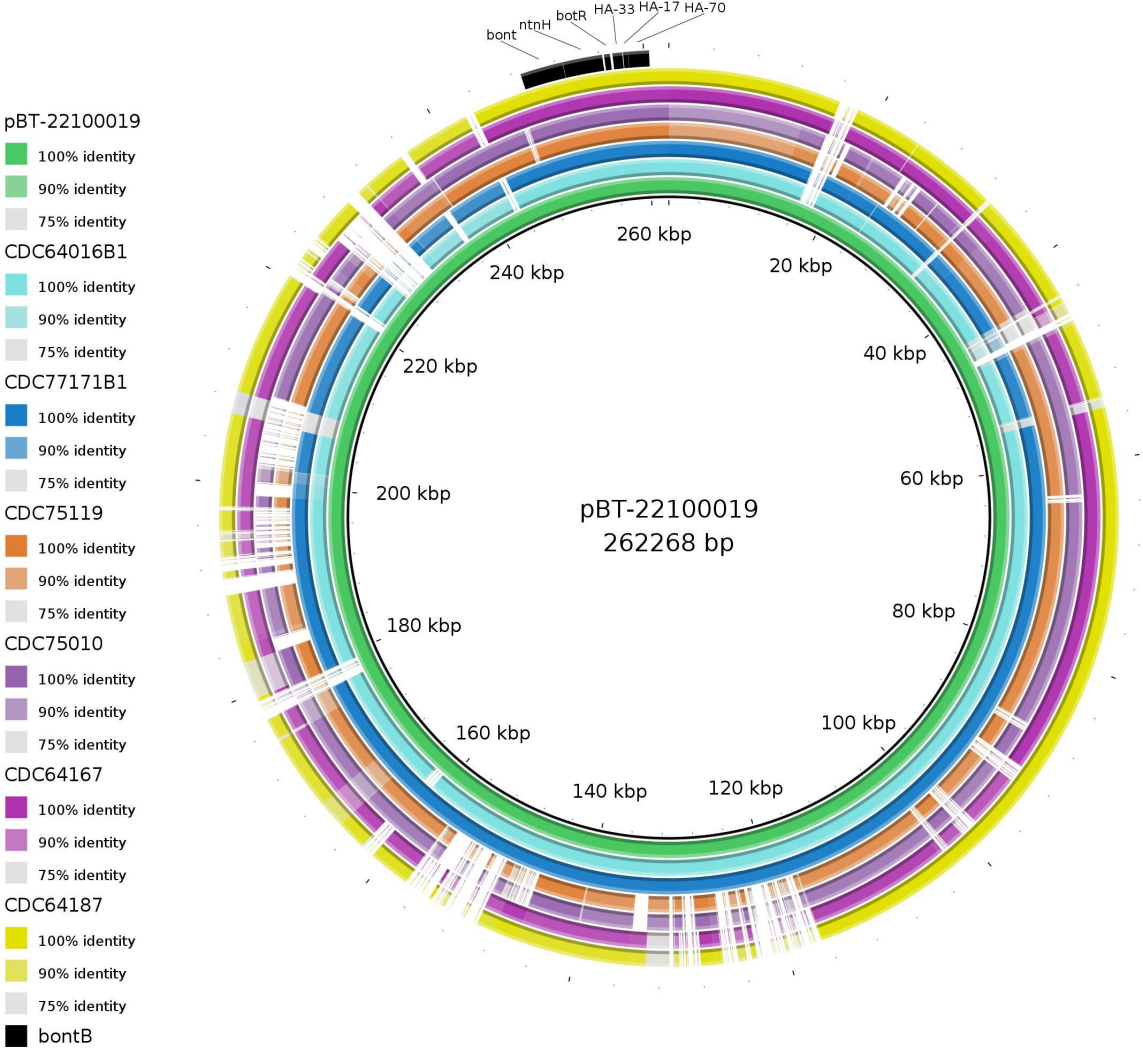
BRIG (Blast Ring Image Generator) comparison of *bont*/B1 carrying plasmid vs. closest putative matche. Sequence homology among four strains and pBT-22100019 (GenBank accession number: CP121697.1). Each ring corresponds to a putative plasmid sequence with the inner ring representing the reference. The color gradient indicates a BLAST result with a matched degree of identity. The *bont*B gene cluster is shown in black.

Characterization of the *bont* gene cluster evidenced that the *bont* gene of all the toxigenic strains was predicted to be located within a plasmid, with an average length of 256,921 bp. blastn results revealed that all 33 toxigenic strains share a >99.7% homology to the plasmid pBT-22100019 (GenBank accession number: CP121697.1, Table S3). Figure 1 depicts the sequence alignment from a subset of strains from our study versus the plasmid match.

Three strains were identified as non-toxigenic by the absence of the *bont* gene cluster; of those strains, NT76259H2 and NT76117PL3 originated from stool specimens that were positive for BoNT/B (data not available for the 3rd non-toxigenic strain). Besides our efforts during the original laboratory investigation, we were unable to recover a toxigenic *C. botulinum* isolate from the sample that originated from NT76117PL3. A *C. botulinum* type B from the sample that originated from NT76259H2 was isolated and sequenced; the *vatD* gene was identified in the non-toxigenic strain only.

### *vatD* gene sequence analysis and comparison

The presence of the *vatD* gene was consistently detected in all 36 isolates across the two different tools and four databases used. The acquired ARG was homologous to the *vatD* gene identified in a streptogramin-resistant *Enterococcus faecium* with 93%–95% identity and 94%–96% coverage (strain BM4145, GenBank accession number: NG_048540.1).

**Fig 2:**
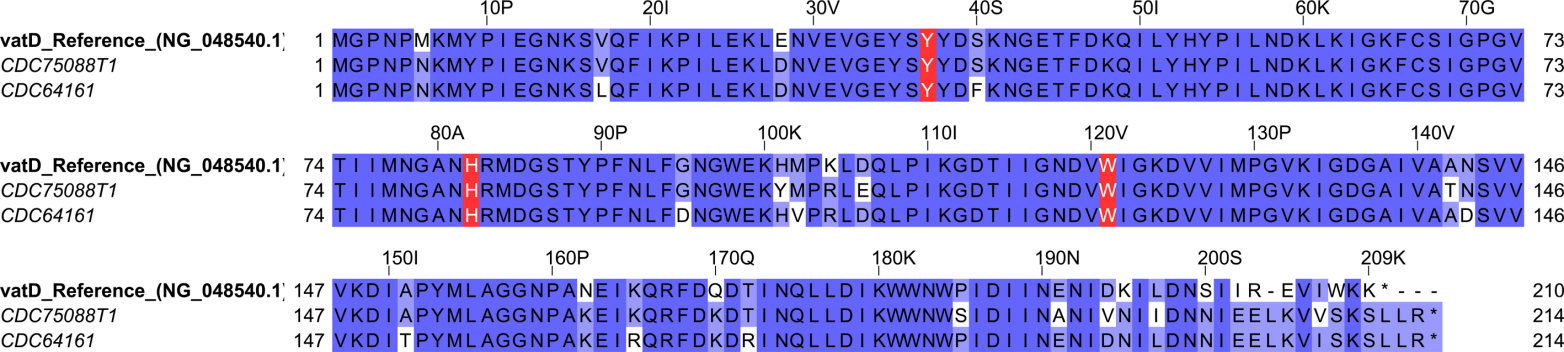
Multiple sequence alignment of the *vatD* amino acid sequences of two representative strains from our study and the reference (GenBank access number: NG_048540.1/WP_002295712). The different shades of purple represent the corresponding amino acid’s similarity (darkest purple tone = 100% similarity). The amino acids highlighted in red are key active site residues, fundamental to the catalytic activity of the *vatD* (Tyr-37, His-82, and Trp-121). Image generated using Jalview v. 2.11.4.1.

Comparative analysis of nucleotide and amino acid sequences revealed notable differences between the *vatD* sequences in our strains and the reference, including amino acid substitutions and nucleotide insertions concentrated toward the end of the sequence (Fig. 2). These changes resulted in the production of a *vatD* protein variant with five additional residues at the C-terminus when compared to the reference (214 aa/209 aa). Despite these variations, the sequences exhibited a high degree of genetic similarities, and the strains from our study maintain a total of 201 amino acids overlap with the highly conserved regions, including key active site residues such as Tyr-37, His-82, and Trp-121 (Fig. S1) (46) with His-82 characterized as a major determinant for the catalytic activity of the *vatD* (47).

Furthermore, multiple sequence alignments of all 36 *vatD*+ strains and the reference evidenced several point mutations across the nucleotide sequences and the presence of two distinct mutation patterns among the samples in our study (Fig. S2).

**Fig 3:**
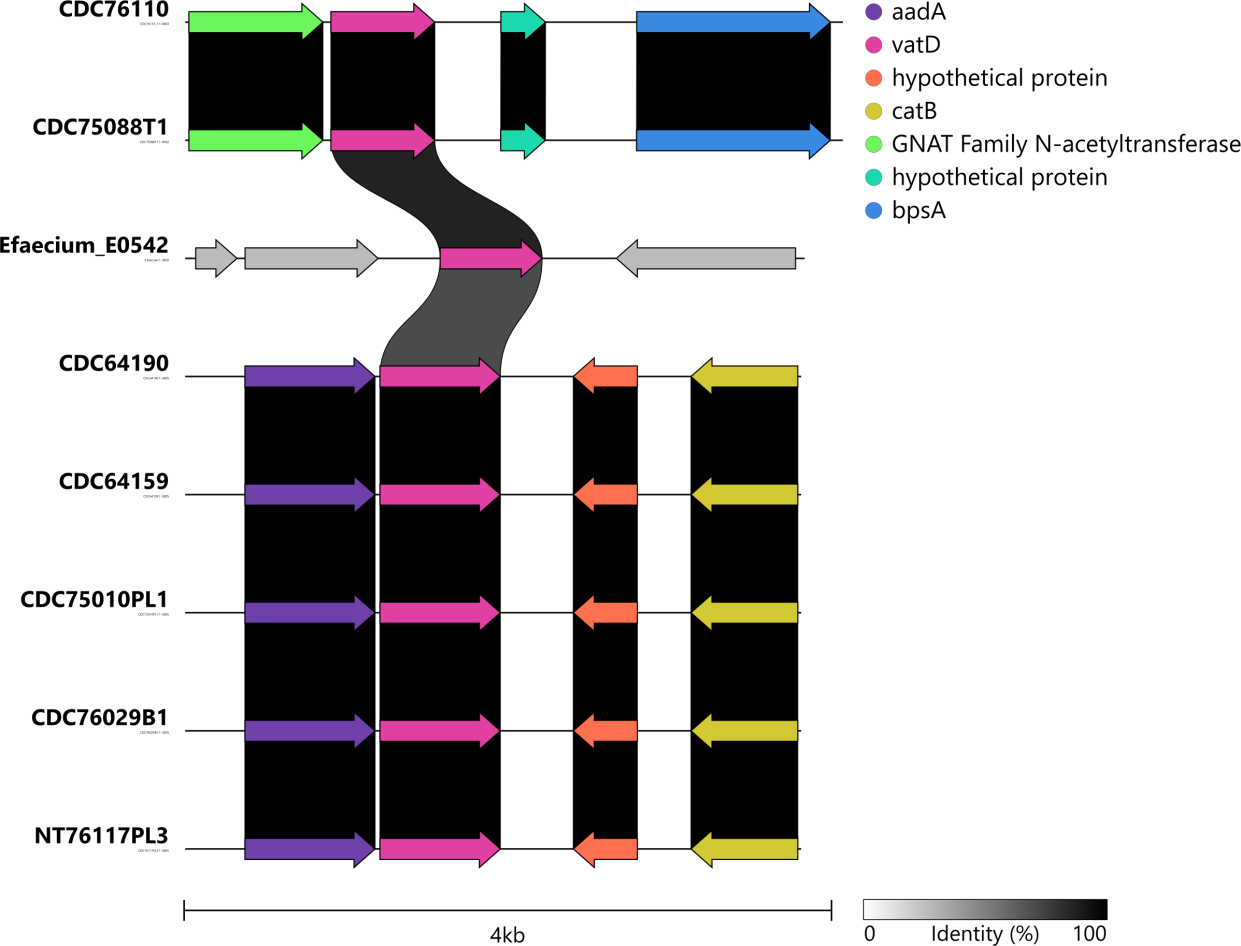
Comparative *vatD* gene cluster analysis among *E. faecium BM4145* and a subset of *C. botulinum* strains harboring the *vatD* gene (pink arrow). Each gene is represented by a color, the direction of the arrows indicates the gene transcription direction, and the sequence identity of linked regions is represented by the shaded gradient. Global alignment and image generation were performed using clinker & clustermap.js v.0.0.25.

Analysis of the *vatD* gene cluster indicated that the gene was located in a contig labeled as chromosomal by MOB-Suite that also carries genes involved in fundamental metabolic functions, such as key enzymes for nucleotide biosynthesis and regulatory post-translational modification. Although no MGEs were identified by the tools used, a search for genomic islands (GI) with IslandViewer showed that in all the strains in our study, the *vatD* gene was located within a predicted GI. Further inspection of the flanking regions revealed the presence of two distinct gene cluster conformations within our strains. As shown in Fig. 3, the *vatD* gene in most of the strains of this study (*n* = 32) is flanked by the genes aadA and catB, which encode antimicrobial resistance genes that usually confer resistance to streptomycin and chloramphenicol drugs, respectively, in *Clostridium species* (48, 49), whereas the remaining strains had bpsA as one of the flanking genes. The presence of flanking genes with high homology to the Clostridium genome rather than those in Enterococcus was universal in all the strains of this study.

**Fig 4:**
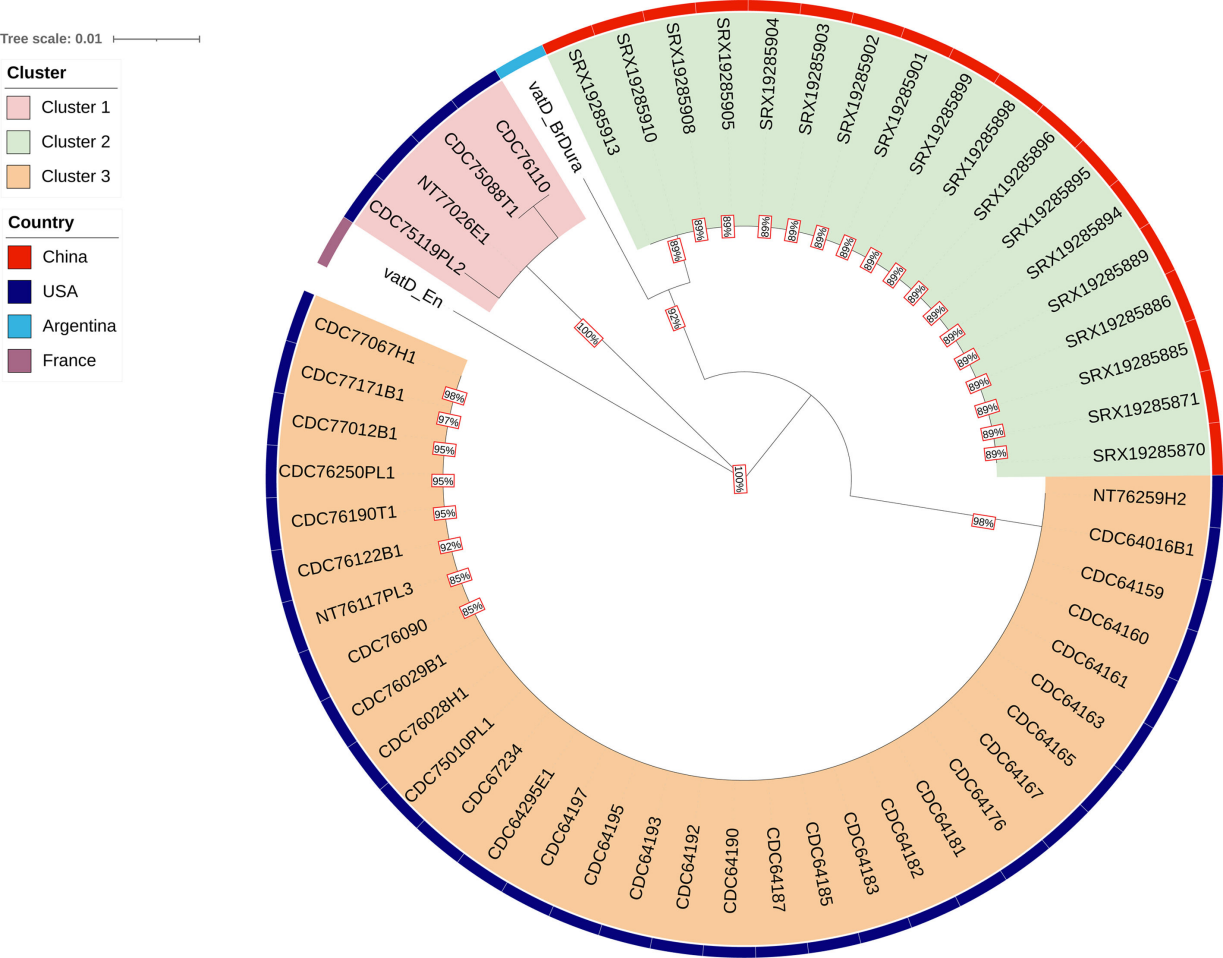
Maximum-likelihood tree based on the amino acid sequences of the *vatD* gene from 55 *Clostridium* strains and the reference, *vatD*_En (GenBank access number: NG_048540.1) as an outgroup. The number at each node represents the bootstrap values. The colored outer ring denotes the country of origin for each strain, and the clusters are indicated by the colored range labels. The tree was generated using MEGA X and visualized with iTOL v6.9.

A maximum-likelihood tree was generated based on the multiple amino acid sequence alignment of the *vatD* sequences of the 36 *vatD*+ strains from this study and 19 *vatD*+ isolates obtained from the NCBI GenBank database (Fig. 4). The *vatD* amino acid sequences from the 55 strains formed three clusters, and the reference formed an outgroup (*vatD*_En, NG_048540.1). Cluster 1 was composed of 4 *vatD* amino acid sequences from strains CDC75088T1, CDC76110, CDC75119Pl2, and NT77026E—the only strains in our study with non-identical *vatD* gene sequences and flanked by the bpsA gene. The Cluster 1 *vatD* amino acid sequences were more closely related to the *vatD* amino acid sequences within strains from China (Cluster 2) than to the other 32 *vatD* amino acid sequences from our study (Cluster 3). Furthermore, the *vatD* sequences from Cluster 2 and Cluster 3 shared a common ancestor with a bootstrap confidence > 80% and had identical *vatD* sequences within their respective clusters. The *vatD* sequence from the Argentinian strain (*vatD*_BrDura) was more closely related to the *vatD* amino acid sequences from China and had the lowest level of homology to the reference.

### Comparative genome analysis

**Fig 5:**
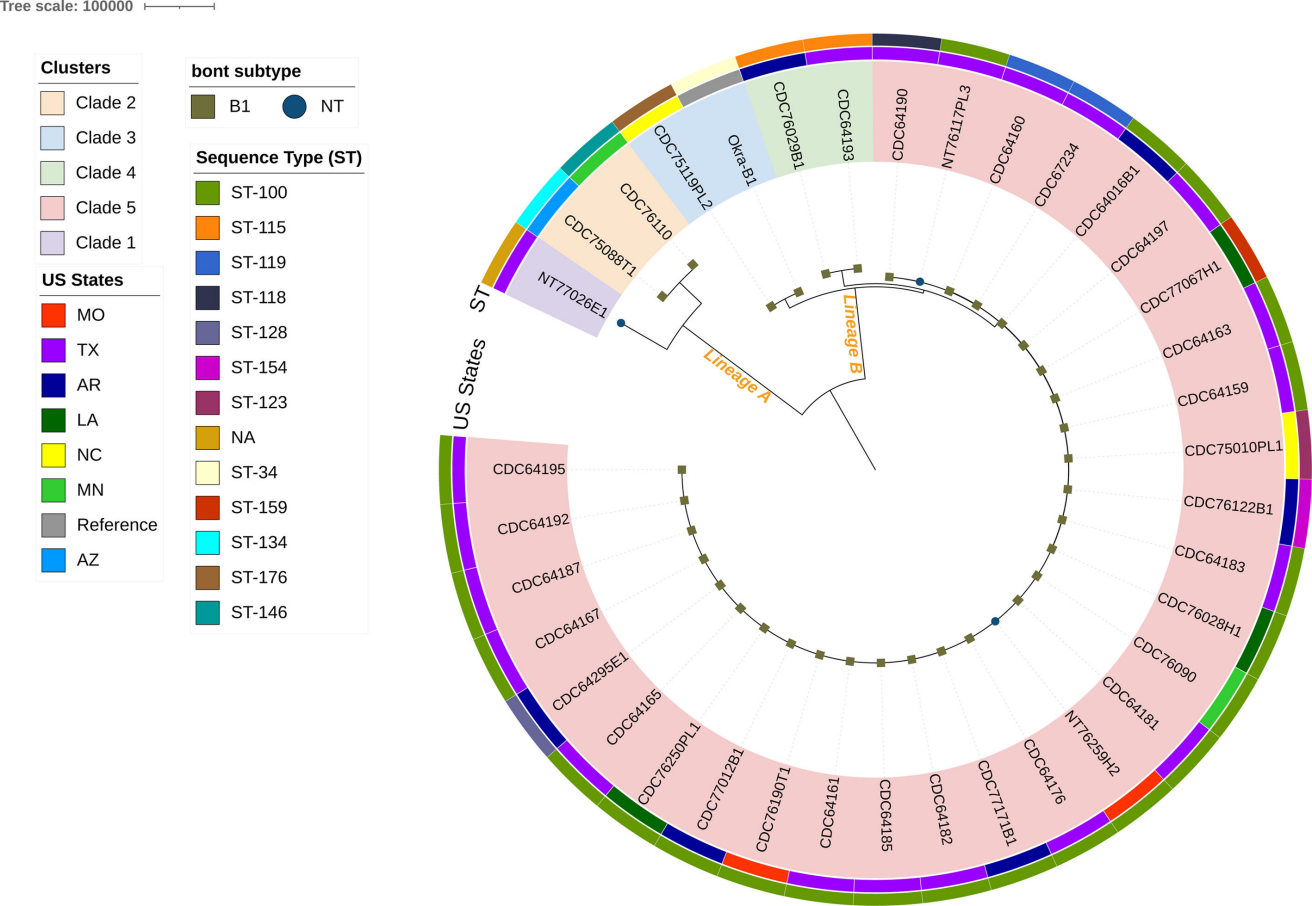
Core genome SNP phylogenetic tree generated using IQ-Tree v.2.2.0.3 and visualized using iTOLv.6.9. SNP analysis was conducted using Snippy against the Okra B1 reference (accession: NC_010516.1). The inner color strip represents the corresponding US states, while the outer ring denotes the corresponding ST. The presence of the *bont*/B1 gene is denoted by a green square at the tips of the tree.

Figure 5 shows the whole-genome sequence phylogenetic tree rooted at the midpoint and built based on a core SNP alignment between the 36 strains from this study and the Okra B1 reference (accession: NC_010516.1). The presence of two lineages, lineage A formed by clades 1 and 2, and lineage B formed by clades 3–5, highlights the diversity among these strains. Strains lacking the *bont* gene were observed in both lineages A (*n* = 1/3) and B (*n* = 2/3). Clade 5 contained the majority of the strains (83%, *n* = 30) and was composed mostly of strains originally from Texas and associated with ST-100. Interestingly, among the 18 Texas isolates within clade 5, only five strains had a median of 79 SNPs difference (range, 74–90), indicating that the isolates from Texas were different strains, despite sharing the same ST and geographical location. Clades 1–3 encompassed the four strains with different mutation patterns in the *vatD* gene sequences. Clade 4 was composed of two strains, CDC76029B1 and CDC64193, originally from Arkansas and Texas, respectively, that share the same ST type, ST-115, and < 130 SNPs of difference. Furthermore, analysis of the core SNPs distance revealed that across Clades 4 and 5, a few strains from Arkansas were more closely related to Texas strains (<200 SNPs) than to the strains within the state (>1,000 SNPs).

**Fig 6:**
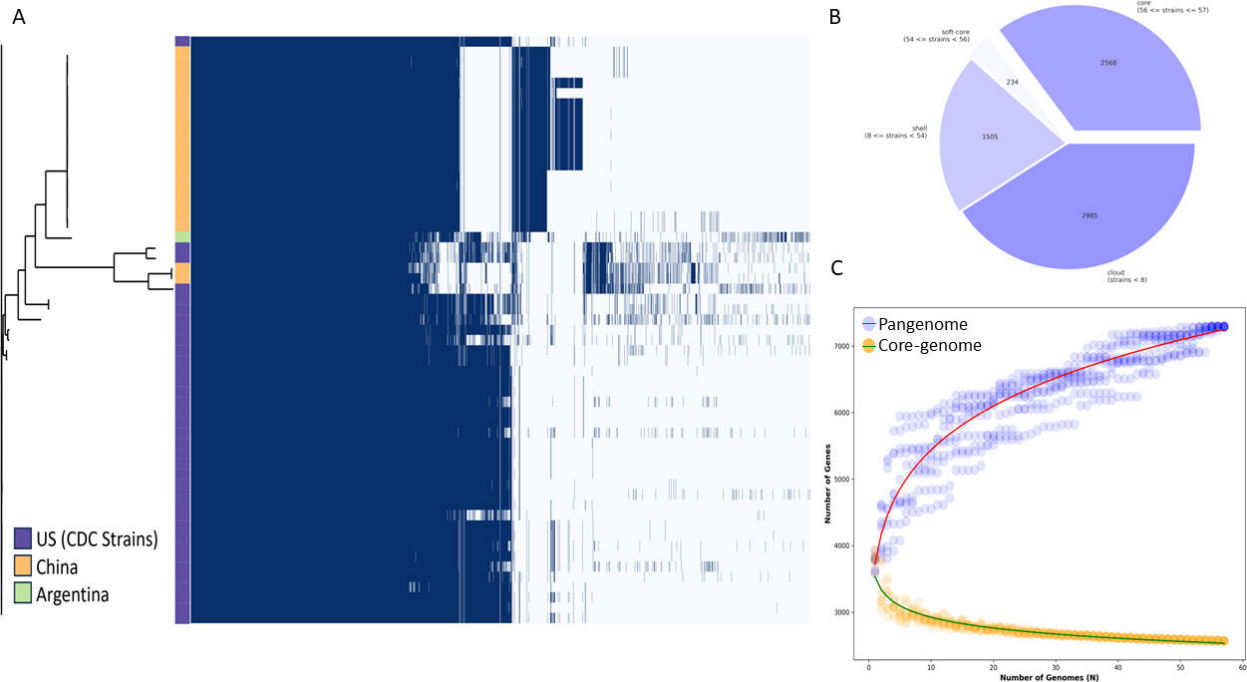
Visualization of the pangenome of *vatD+ Clostridium* strains. Pangenome analysis was performed using Panaroo. (**A**) Pangenome maximum likelihood tree compared to a matrix with the presence (blue) and absence (white) of core and accessory genes, purple represents the strains from our study (US CDC strains), yellow the strains from China, and light green the strain from Argentina. (**B**) Pie chart with the breakdown of the number of genes in the pangenome. (**C**) Pan-genome (light purple/red) versus core genome (yellow plot/green) plot to indicate the openness of the *C. botulinum* pangenome. The pangenome trajectory increases as more genomes are added to the analysis.

Using Panaroo, the pangenome of all the *vatD*+ *C. botulinum* strains (36 from our study and 19 downloaded from NCBI) was generated. A total of 7,292 annotated genes were identified; of these, 2,568 were present in all genomes (core genes), 234 genes were present in 95%–99% of the genomes (soft-core genes), 1,505 in 15%–95% (shell genes), and 2,985 genes in <15% of the genomes (cloud genes) (Fig. 6B). Further analysis estimated that the pangenome of *C. botulinum* is open, with a Heaps’ Law gamma coefficient of 0.16, and as depicted by the progressive increase of the pangenome size as more genomes are added (Fig. 6C). Although the distribution of accessory genes varied among the strains, Fig. 6A highlights the presence of clusters based on the gene presence/absence matrix, with the majority of the strains from our study clustering together at the bottom of the tree.

## DISCUSSION

In this study, we described the molecular characterization of 33 *C*. *botulinum* and 3 related non-toxigenic strains recovered from infant botulism cases harboring an unusual ARG, the *vatD* gene. To the best of our knowledge, this is the first report of the detection of the *vatD* gene in the chromosome of *C. botulinum* strains recovered from infant botulism cases. Due to the limited clinical relevance, phenotypic and genotypic patterns of antimicrobial resistance have not been extensively studied in *C. botulinum* strains, and little is known about the occurrences and location of ARGs in the genome of the bacterium.

The presence of the *vatD* gene (formerly known as satA) was first described on a streptogramin-resistant *E. faecium* (SREF), strain BM4145, recovered from a clinical sample in Europe in 1984 (40). Subsequently, several studies reported the presence of SREF isolates harboring the *vatD* gene in both animal and human sources in Denmark (50), the Netherlands (16), Germany (51), and Western Europe (52). These studies also established a link between the use of streptogramin antibiotics, particularly virginiamycin, as growth promoters in farm animals, and the increased detection of SREF isolates and transmission within the community. These findings led the European Union to ban the usage of virginiamycin as a growth promoter due to the increased concern of cross-resistance to quinupristin-dalfopristin (Q/D) (15–17), a semisynthetic streptogramin antibiotic once used as a last resource to treat vancomycin-resistant *Enterococcus faecium* (13, 53).

Similarly, a few studies in the US have also shown an increased correlation between the usage of virginiamycin in poultry and the detection of Q/D-resistant *E. faecium* isolates (54–56). However, differently than what was observed in Europe, the detection of Q/D-resistant *E. faecium* isolates recovered from humans is relatively rare in the US (57), and the use of streptogramin antibiotics in farm animals for growth promotion and for prevention of necrotic enteritis through control of *Clostridium perfringens* remains an option. Furthermore, molecular studies of SREF or Q/D–resistant *E. faecium* isolates recovered from human and non-human sources revealed that the *vatD* gene is rarely detected in those isolates. To date, only two papers have reported the detection of *vatD*+ *E. faecium* isolates in poultry (18) and in a clinical specimen (58) in the US.   

The discovery of the *vatD* gene in *C. botulinum* strains recovered from clinical specimens is intriguing, despite its low prevalence in the US. Furthermore, analysis of the nucleotide sequences of the *vatD* gene in our strains showed a high homology (93%–95% identity) to the *vatD* from *E. faecium* strain BM4145. This observation is not unique to our research; a study on chicken carcasses in the US, conducted by Jackson and collaborators, found that all *vatD* gene sequences (*n* = 3) identified in enterococci isolates were identical to BM4145 and each other (18). In comparison, in our study, 32 strains carried an identical *vatD* gene, whereas four strains showed slight variations, ranging from zero to five nucleotides amongst themselves, and up to 40 nucleotides when compared to the other 32 strains. Interestingly, pairwise identity analysis revealed that the non-identical *vatD* sequences were more closely related to the *vatD* on strain BM4145, highlighting the possibility of distinct evolutionary events.

The mutations observed on the *vatD* gene in all the strains in our study raise the question of the functionality of this gene in eliciting a phenotypic antimicrobial resistance activity. Analysis of the open reading frame reveals that the translated protein exhibits a longer sequence when compared to the reference sequence (214aa/209aa), with five additional amino acids at the C-terminus. Although the protein sequence of the strains in our study shows a total of 201 amino acids overlapping with the reference, the less than 100% identity (93%–95% identity and 94%–96% coverage) and the point mutations and insertions suggest that the resistant mechanism for this gene might be altered. However, without phenotypic assays to confirm the expression and activity of this gene, the functionality of the *vatD* gene in the strains in our study remains uncertain.

Although no AST test was conducted, analysis of the protein sequences revealed that key active site residues were highly conserved across the gene, including His-82, a major determinant for the *vatD* gene activity. For instance, a study conducted by Kehoe and collaborators in 2003 demonstrated that replacing the His-82 residue resulted in a fivefold reduction of the *vatD* catalytic activity (47). While we cannot infer enzymatic activity solely based on the presence of these residues, the detection of His-82 in our strains’ *vatD* genes suggests the potential for catalytic activity that warrants further investigation, particularly regarding the phenotypical expression of the resistance.

In the US, most IB cases are associated with *C. botulinum type B*. Out of the 226 isolates sequenced between 2020 and 2023, 127 were positive for serotype B, 98 of those harbored a bont/B1 gene*,* and 33 of these also carried a *vatD* gene variant. We predict that the three *vatD*+ non-toxigenic strains might have originally carried a bont/B1 gene in a plasmid, which was subsequently lost during the isolation and cultivation process. This hypothesis is corroborated by (i) two of the three non-toxigenic strains were associated with a confirmed *C. botulinum* type B case and (ii) all 33 toxigenic strains carried the bont/B1 gene on a putative plasmid. We note that not all *C. botulinum bont*/B1 strains carry a *vatD* gene; for instance, in two isolates from the same specimen, CDC76259H1 (*bont*/B1) and CDC76259H2 (non-toxigenic), the *vatD* gene variant was present in the non-toxigenic strain and absent in the toxigenic pair. The reasons that led to this genomic event remain unclear, and additional research is needed to elucidate the genetic background of these strains.

A definitive source of *C. botulinum* spores is rarely implicated in IB cases; however, studies have shown that infants living in rural/farm environments or who have been exposed to soil from active construction sites might have a higher risk of contracting botulism (59–61). Though we do not have information on source attribution for the cases in our study, it is interesting that the two states with the highest number of isolates carrying a *vatD* gene variant, Texas (*n* = 20) and Arkansas (*n* = 6), are also listed as leading states for livestock production (https://quickstats.nass.usda.gov/ last access July 2024). This includes cattle and poultry industries where the use of virginiamycin is permitted. These findings suggest a potential parallel to European studies in which streptogramin use in livestock was linked to the detection of *vatD*+ isolates in the community; here, we noted the possible emergence of *vatD+ C. botulinum* strains instead.

It is important to note that in the US, while the use of the streptogramin Q/D to combat vancomycin-resistant *Enterococcus faecium* has been discontinued since 2022, the veterinary formulation, virginiamycin, remains approved for use in food animals. Taking into consideration that the majority of our strains were collected before 2022, it would be particularly interesting to investigate whether the discontinuation of Q/D antibiotics in clinical settings, coupled with the continued use of virginiamycin in agriculture, leads to any changes in the incidence of *C. botulinum* strains carrying the *vatD* gene.

A question that remains to be answered is “What is the origin of the *vatD* gene within the genome of *C. botulinum?”* Naturally, the Clostridium genus is well known for its capability of genetic exchange as evidenced by the presence of the *bont* gene cluster within *C. botulinum* species and among other clostridia, such as *C. baratii* and *C. butyricum* (20, 62–64). The ubiquitous and overlapping presence of both *C. botulinum* and *E. faecium* in the environment, coupled with the high sequence homology between their *vatD* genes and the detection of a *bont*-like gene in an *E. faecium* isolated in cow feces (23), hints at the possibility of ongoing genetic exchange between the species. Analysis of the *vatD* gene composition and flanking regions suggests an earlier integration of the *vatD* gene into the genome of *C. botulinum* species. This hypothesis is supported by the low GC composition (29.8%) of the *vatD* gene and the presence of flanking genes exhibiting high homology to those found in Clostridium rather than Enterococcus genomes, along with the putative placement of the v*atD* gene cluster within a GI that harbors other virulence factors. Furthermore, the discovery of similar *vatD* sequences in *C. botulinum* strains from diverse sources and BoNT types found in China and Argentina adds to the pool of evidence of an earlier genomic exchange. An examination of publicly available strains from these countries, 18 from China and 1 from Argentina, revealed a consistent *vatD* gene cluster and a shared adjacent gene, aadA, which was also present in all but four of our studied strains, corroborating to the evidence of distinct evolutionary events for the *vatD* gene variant in the *C. botulinum* strains in our study.

The findings reported in this study highlight the importance of integrating WGS technologies and bioinformatics tools into a laboratory workflow to enhance our understanding of the diversity and plasticity of the *C. botulinum* genome. Furthermore, the data presented here provide supplemental evidence of ongoing genetic exchange between species within the Firmicutes phylum, from toxins to antimicrobial resistance genes. However, further studies are necessary to comprehend the evolutionary origin of this *vatD* gene in the genome of *C. botulinum* and to determine whether this gene confers phenotypical resistance. Ultimately, these investigations will be crucial for advancing our knowledge of *C. botulinum* and its impact on public health.

## Data Availability

The reads and genome sequences have been deposited in NCBI at the SRA and GenBank as noted under the Bioproject accession number PRJNA428620.

## SUPPLEMENTAL MATERIAL

Supplemental figures aem.00090-25-s0001.pdf Figures S1 and S2.

Supplemental tables aem.00090-25-s0002.xlsx Tables S1 to S3.

ASM does not own the copyrights to Supplemental Material that may be linked to, or accessed through, an article. The authors have granted ASM a non-exclusive, world-wide license to publish the Supplemental Material files. Please contact the corresponding author directly for reuse.
