# Supplementary material for: Detection of a streptogramin A O-acetyltransferase gene (vatD) in the chromosome of Clostridium botulinum isolated from infants in the United States
Source: Appl Environ Microbiol. 2025 Jul 22;91(8):e00090-25. doi: 10.1128/aem.00090-25 (PMC12366311; doi:10.1128/aem.00090-25)

Supplementary Fig S1. Multiple sequence alignment of the amino acid sequences of the vatD in all the strains and the reference. The amino acid mismatches are denoted by the colored letters and presence of the key active sites, Tyr-37, His-82, and Trp-121, are highlighted by the red box.

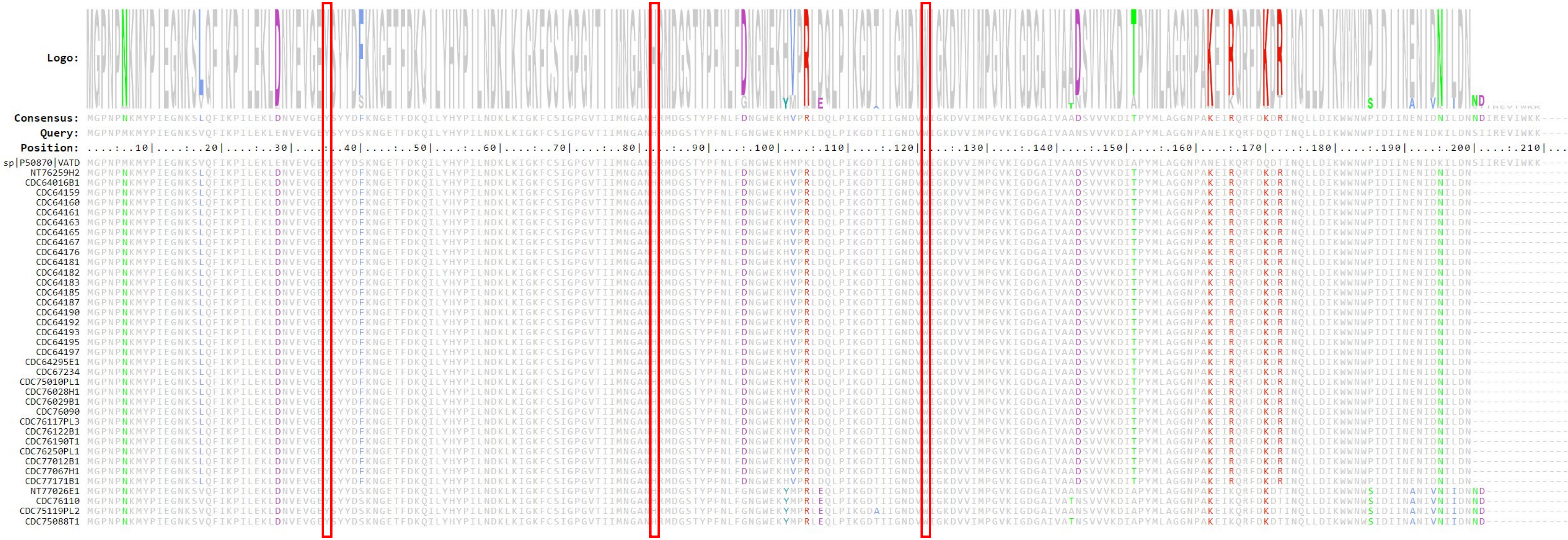

Supplementary Fig. S2. Multiple sequence alignment of the vatD nucleotide sequences from all the strains and the reference (GenBank access number: NG_048540.1). Sequences were aligned using MUSCLE algorithm via Geneious Prime. Different color patterns denote nucleotide mismatch (Red = A; Green = T; Blue = C, and Yellow = G). The strains highlighted by the red box harbor vatD nucleotide sequences that differ from the remaining str[illegible]

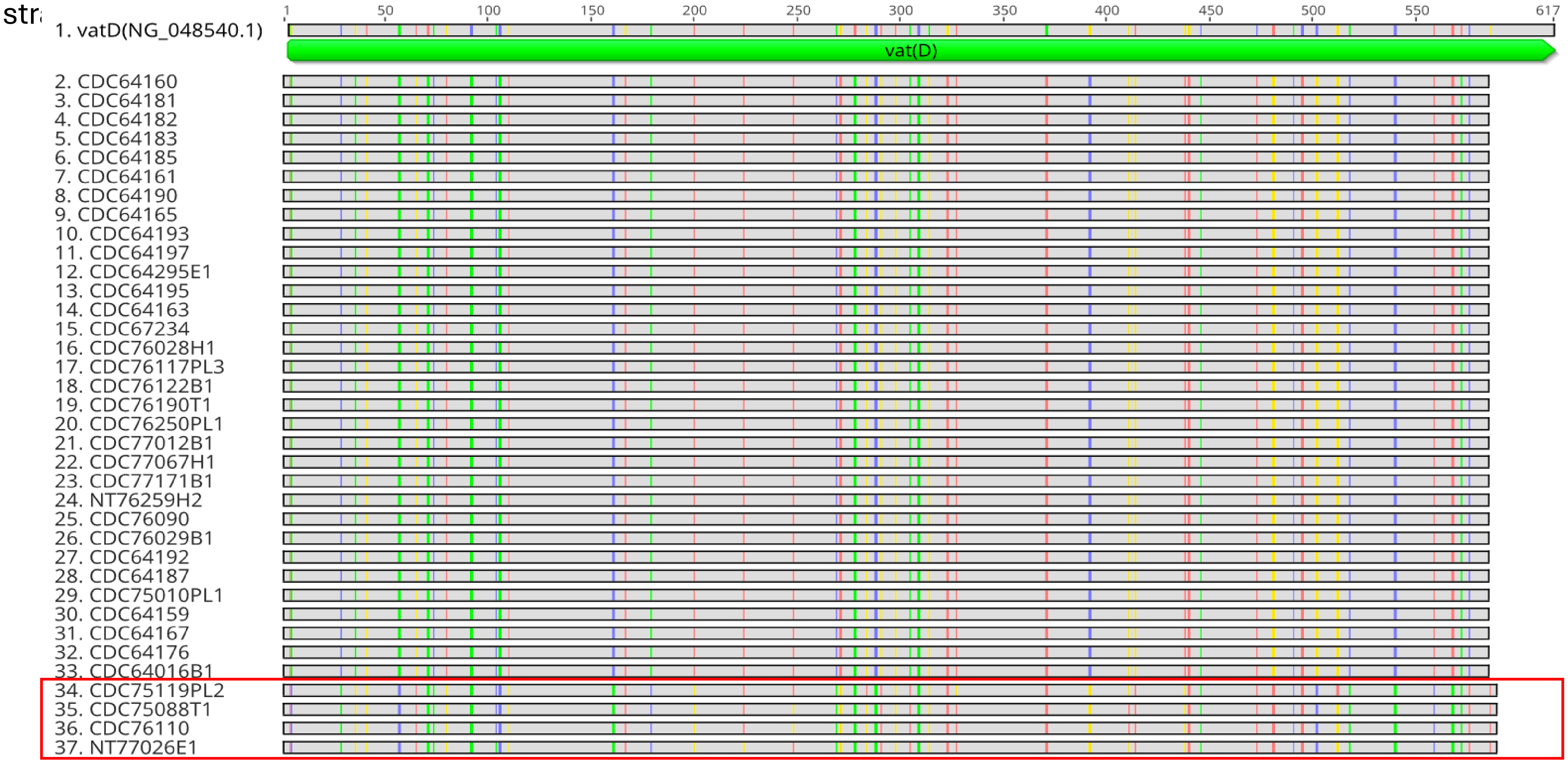

Supplement: Supplemental figures — Figures S1 and S2. [file aem.00090-25-s0001.pdf]
